# NK-92 cells labeled with Fe_3_O_4_-PEG-CD56/Avastin@Ce6 nanoprobes for the targeted treatment and noninvasive therapeutic evaluation of breast cancer

**DOI:** 10.1186/s12951-024-02599-x

**Published:** 2024-06-05

**Authors:** Jingge Lian, Meng Li, Meng Duan, Yaqian Sun, Zilin Wang, Xinyu Guo, Jingchao Li, Guo Gao, Kangan Li

**Affiliations:** 1 Department of Radiology, Songjiang Hospital Affiliated to Shanghai Jiaotong University School of Medicine, Shanghai, 201600 P.R. China; 2 Department of Radiology, Peking University Third Hospital, Beijing, 100191 China; 3 State Key Laboratory for Modification of Chemical Fibers and Polymer Materials, College of Biological Science and Medical Engineering, Donghua University, Shanghai, 201620 China; 4 Department of Instrument Science and Technology, School of Electronic Information and Electrical Engineering, Shanghai Jiao Tong University, Shanghai, 200240 China; 5 Department of Immunology, School of Cell and Gene Therapy, Songjiang Research Institute, Shanghai Jiao Tong University School of Medicine, Shanghai, 201600 P.R. China

**Keywords:** Multimodal nanomaterials, NK-92 cells, Magnetic resonance imaging, Adoptive cellular immunotherapy, Photodynamic therapy

## Abstract

Adoptive cellular immunotherapy as a promising and alternative cancer therapy platform is critical for future clinical applications. Natural killer (NK) cells have attracted attention as an important type of innate immune regulatory cells that can rapidly kill multiple adjacent cancer cells. However, these cells are significantly less effective in treating solid tumors than in treating hematological tumors. Herein, we report the synthesis of a Fe_3_O_4_-PEG-CD56/Avastin@Ce6 nanoprobe labeled with NK-92 cells that can be used for adoptive cellular immunotherapy, photodynamic therapy and dual-modality imaging-based in vivo fate tracking. The labeled NK-92 cells specifically target the tumor cells, which increases the amount of cancer cell apoptosis in vitro. Furthermore, the in vivo results indicate that the labeled NK-92 cells can be used for tumor magnetic resonance imaging and fluorescence imaging, adoptive cellular immunotherapy, and photodynamic therapy after tail vein injection. These data show that the developed multifunctional nanostructure is a promising platform for efficient innate immunotherapy, photodynamic treatment and noninvasive therapeutic evaluation of breast cancer.

**Supplementary Information:**

The online version contains supplementary material available at 10.1186/s12951-024-02599-x.

## Introduction

In women, breast cancer is the most common type of cancer and the leading cause of cancer-related death [1–3]. Conventional treatment for breast cancer includes surgical resection, radiotherapy, chemotherapy and hormone therapy [4–6]. Patients with aggressive forms of this disease have significantly shortened disease-free survival and overall survival (OS) and increased resistance to radiotherapy and chemotherapy [7–9]. Currently, adoptive cellular immunotherapy has developed into a clinically validated therapy for many diseases, especially cancer [10, 11]. Immune cell-mediated cancer therapy has been actively investigated and exhibits promising antitumor efficacy in both hematological cancers and solid tumors, with five chimeric antigen receptor (CAR) T cells have been approved by the U.S. Food and Drug Administration (FDA) [12–16]. Compared with T cells, Natural kill (NK) cells have distinctive mechanisms to target and eliminate cancer cells, including the capacity to destroy tumor cells through antibody-dependent cell-mediated cytotoxicity (ADCC), available cell line for adoptive transfer in humans to decrease therapeutic cost [17–20], and the approved clinical uses of NK-92 cells by the FDA. However, the treatment effects of these cells for solid tumors are significantly worse than those on hematological tumors, which may be due to certain limitations, such as inadequate targeting and an insufficient local concentration of NK cells in the tumor area.

In addition, photodynamic therapy (PDT) has been recently adopted as a treatment strategy, which can be achieved by applying photosensitizers and light in combination with oxygen to produce reactive oxygen species (ROS) for destroying tumor cells [21–26]. Furthermore, PDT can synergistically boost the efficiency of immunotherapy through inducing the immunogenic cell death [27–30]. Many types of photosensitizers have been used for PDT, and Chlorin e6 (Ce6) is a second-generation photosensitizer that can be used to treat various cancers [31–35]. In addition to PDT effect, Ce6 also absorb light at the long wavelengths (660 nm) and thus can be used as an ideal fluorescent label for optical imaging [36, 37]. Therefore, the combination of PDT with immunotherapy with the imaging capability using Ce6-based probe has the potential to be applied in cancer treatment.

The use of imaging technologies to guide therapy and evaluate therapeutic outcome is important to increase the treatment efficacies. Magnetic resonance (MR) imaging is one of the most commonly used non-invasive imaging techniques in clinical practice with the advantages of high spatial resolution, strong tomographic imaging capabilities and negligible penetration depth limitation [38]. Optical imaging, such as fluorescence imaging often can achieve diagnosis with very high sensitivity and spatial resolution [39]. However, each imaging mode has certain limitations and shortcomings and thus fails to provide accurate diagnostic information independently. The combination of two or more imaging modes can improve the accuracy of imaging [40].

**Fig. 1:**
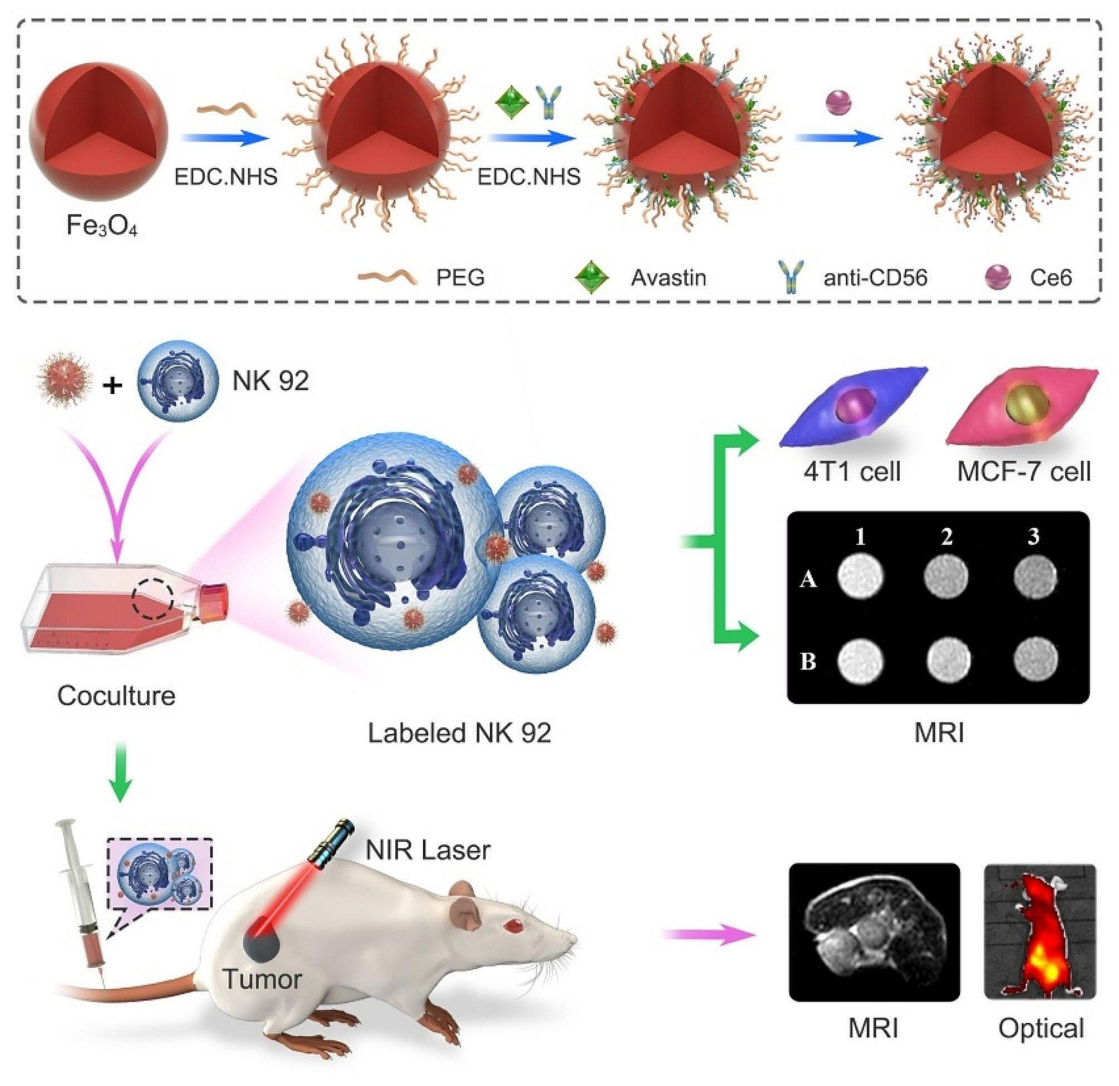
Schematic of the synthesis of Fe_3_O_4_-PEG-CD56/Avastin@Ce6 nanoprobe and their use for PDT-immunotherapy and MR/optical dual molecular imaging

Herein, Fe_3_O_4_ nanoparticles (NPs) were coated with a layer of polyethylene glycol (PEG) to improve biocompatibility [22], and the surface of these PEGylated Fe_3_O_4_ NPs (Fe_3_O_4_-PEG-COOH) were further decorated with bevacizumab (Avastin®), anti-CD56 antibody and Ce6 to form a nanocomposite (Fe_3_O_4_-PEG-CD56/Avastin@Ce6). Bevacizumab, a clinic available monoclonal immunoglobulin G antibody targeting VEGF can inhibit blood vessel growth and has a direct effect on tumor cells, contributing to nanoprobe targeting of breast cancer cells [41–43]. Meanwhile, due to the high expression of CD56 on the surface, NK-92 cells can be stably and specifically labeled by the probe in a short period of time [44–47]. Therefore, the formed Fe_3_O_4_-PEG-CD56/Avastin@Ce6 NPs were synthesized to label NK-92 cells for their targeted delivery to breast cancer cells, which would facilitate targeted therapy and non-invasive imaging of breast cancer by NK-92 cell. Previous study has indicated that the labeling of NK cells with nanoparticles will not affect their functions [48]. In this study, NK-92 cells labeled with Fe_3_O_4_-PEG-CD56/Avastin@Ce6 were used for in vitro and in vivo breast cancer adoptive cellular immunotherapy and PDT as well as MR/optical dual-modal imaging (Fig. 1).

## Materials and methods

### Materials

FeSO_4_·7H_2_O and ferric citrate were provided by Sinopharm Chemical Reagent Co., Ltd. (Shanghai, China). Amine-PEG_2k_-carboxyl was purchased from Shanghai Yare Biotechnology Co., Ltd. (Shanghai, China). Hoechst 33,342 was purchased from Sigma-Aldrich Chemical Co., Ltd. (St Louis, MO, USA). N-Hydroxysuccinimide (NHS), 1-(3-dimethylaminopropyl)-3-ethylcarbodiimide hydrochloride (EDC) and Ce6 were obtained from Aladdin Chemical Reagent Co., Ltd. (Shanghai, China). Cell culture medium, horse serum, fetal bovine serum (FBS), and penicillin-streptomycin were purchased from Gibco (USA). Recombinant IL-2 was obtained from PeproTech (USA). Bevacizumab (Avastin®) was obtained from Roche (Genentech, South San Francisco, CA). The anti-CD56 antibody was obtained from BD (USA). The CCK-8 agent was purchased from NCM Biotech Co., Ltd. (Suzhou, China).

### Synthesis of targeted NPs

Fe_3_O_4_ NPs were produced according to our previous study [49]. After that, EDC and NHS were separately mixed with sodium borate buffer, pH = 9 (SBB 9) (3.125 mg/mL), followed by the addition of 0.1 mL of Fe_3_O_4_-COOH NPs. Then, 10 mg of PEG was dissolved in 100 µL of deionized water and mixed well with 0.3 mL of the SBB 9/Fe_3_O_4_-COOH NPs mixture, which was maintained at room temperature for 12 h. After the reaction was finished, the product solution was purified with an ultrafiltration device (Millipore, Mw = 100 kDa) for 10 min at 2000 r.p.m., and the product (Fe_3_O_4_-PEG-COOH) was collected. To activate the carboxyl group on the Fe_3_O_4_-PEG NPs, EDC and NHS (200 µL, 6.25 mg/mL) were added for 20 min of incubation. Then, 50 µL of CD56 and 50 µL of Avastin were added to the activated Fe_3_O_4_-PEG NPs to allow 12 h of reaction. Next, the product solution was purified with an ultrafiltration device (Millipore, Mw = 100 kDa). Then, 500 µL of a 5 mg/mL solution of Ce6 dissolved in 0.01 M NaHCO_3_ (pH 8.3) was added into the aforesaid concentrated solution, and the mixture was left at 37 °C for 24 h on a shaking bed. Finally, the product was collected and dialyzed against water in a dialysis bag (MWCO = 3500) for three days with water changing every four hours. The samples were kept at 4 °C for further use.

### Characterization techniques

The morphology and size of the Fe_3_O_4_-PEG-CD56/Avastin@Ce6 nanoprobes were examined by Transmission electron microscopy (TEM, JEM-2100 electron microscope, JEOL, Japan). One hundred NPs were randomly selected and analyzed by ImageJ software. TGA of the Fe_3_O_4_-PEG-CD56/Avastin@Ce6 NPs were acquired by a Thermo gravimetric analyzer (Pyris 1 TGA, Perkin Elmer, America) under a N_2_ atmosphere at a heating rate of 20 °C/min. The UV-visible absorption spectrum was acquired with a Varian Cary 50 spectrophotometer. FTIR spectroscopy was performed with a PerkinElmer GX spectrophotometer. The T2 relaxation times of increasing concentrations of the nanoprobe were measured with a 3.0 T MRI system.

### Cell culture

4T1 cells and MCF-7 cells were cultured in RPMI-1640 supplemented with 10% FBS and 1% penicillin-streptomycin at 37 °C with 5% CO_2_. The base medium for the NK-92 cell line was α-MEM with 2 mM L-glutamine and 1.5 g/L sodium bicarbonate and without the addition of ribonucleosides and deoxyribonucleosides. The following components were added to the base medium to make complete growth medium: 0.2 mM inositol, 0.1 mM 2-mercaptoethanol, 0.02 mM folic acid and 100–200 U/mL recombinant IL-2; additionally, the final concentrations of horse serum and FBS were adjusted to 12.5% and 12.5%, respectively. Cells were cultured in complete growth medium at 37 °C with 5% CO_2_.

### Cytotoxicity evaluation

A CCK-8 cell viability assay was used to determine whether the NPs cause adverse effects on the biological characteristics of NK-92 cells. Briefly, NK-92 cells were cultured overnight in 96-well plates. Then, 100 µL of culture medium containing different concentrations of the nanoprobes was added for incubation for an additional 12 h, 24–48 h. Next, 20 µL of CCK-8 reagent were added. After incubation for 3 h, the optical density (OD) values were obtained at 450 nm using a microplate reader. Cell viability was determined according to the following formula: cell viability (%) = (A_experimental group_ − A_blank_)/ (A_control group_− A_blank_) × 100%. The values from triplicate wells were measured for each group. In addition, the cellular activity of NK-92 cells after the treatment with nanoprobes during laser was also assessed by using CCK-8 assay. FCM was performed to evaluate cell apoptosis and the cell cycle after incubation with different concentrations of Fe_3_O_4_-PEG-CD56/Avastin@Ce6 NPs. For cell apoptosis analysis, labeled NK-92 cells were washed 3 times with binding buffer. 5 µL of FITC-annexin V and 100 µL of binding buffer were added to the cells. After 15 min of incubation in the dark, 5 µL of propidium iodide (PI) solution and 400 µL of binding buffer were added to each tube. Finally, the fluorescence signal was detected by FCM. For cell cycle analysis, the labeled NK-92 cells were fixed in 75% ethanol for 24 h, the cells were stained with PI and RNase (BD) and then washed with PBS. The cell cycle was analyzed by measuring the DNA content by FCM.

### Measurement of reactive oxygen species

ROS production was measured using 2’,7’-dichlorofluorescein diacetate (DCFH-DA). NK-92 cells labeled with Fe_3_O_4_-PEG-CD56/Avastin@Ce6 were incubated with the target cells for 24 h at 37 °C (NK + NPs). The NK + NPs + L group were irradiated with a laser for 15 min at 6 h, 12 h and 24 h. Next day, the cells were washed twice with PBS and incubated with DCFH-DA in a final concentration of 10 µM for 30 min. Cells were rinsed with PBS and then treated with H_2_O_2_ for 30 min. DCF fluorescence intensity was measured at excitation wavelength of 485 nm and emission wavelength of 535 nm.

### Cell labeling and NP localization

NK-92 cells were incubated with Fe_3_O_4_-PEG-CD56/Avastin@Ce6 (10 µg/mL) at 37 °C for 24 h in complete medium. Labeled NK-92 cells were purified by density-gradient centrifugation, and the unincorporated NPs were removed from the solution. The sample was washed with PBS and fixed with electron microscope fixative. To determine whether the NPs were internalized or mainly adsorbed on the cell surface, NP localization was observed by TEM. In addition, confocal laser scanning microscope (CLSM) was used to evaluate the cellular uptake. Labeled NK-92 cells were washed with PBS twice and incubated with fresh medium. Then the lysosomes were stained with Lyso-Tracker Green dye for 45 min and the cell nucleus were stained with Hoechst33342 for 15 min. The photographs of stained cells were collected by CLSM (Zeiss 800). FCM was used to examine cellular uptake efficiency. NK-92 cells were seeded in 24-well cell culture plates at a density of 2 × 10^5^ cells per well and then cultured in fresh complete medium containing Fe_3_O_4_-PEG-CD56/Avastin@Ce6 or Fe_3_O_4_-PEG-Avastin@Ce6 at concentrations of 0, 5, 10, 15 and 20 µg/mL. After 4 h of incubation, the cells were washed with PBS 3 times and resuspended in 500 µL of PBS for FCM analysis on a Becton Dickinson FACScan analyzer. The FL3 fluorescence of 10,000 cells was measured, and the mean fluorescence intensity of the gated viable cells was quantified.

### MR and optical imaging of NK-92 cells in vitro

NK-92 cells were cultured in fresh complete medium containing Fe_3_O_4_-PEG-CD56/Avastin@Ce6 or Fe_3_O_4_-PEG-Avastin@Ce6 at concentrations of 0, 10 and 20 µg/mL. After 4 h of incubation, the cells were washed with PBS and then resuspended in 500 µL of 1% agarose. MRI was performed using a 3.0 T MRI system with a wrist coil. T2WI were acquired using the following scanning parameters: repetition time (TR) = 2500 ms, echo time (TE) = 60 ms, field of view (FOV) = 130 mm×104 mm, slice thickness = 3 mm, and slice gap = 0.1 mm. The T2 signal intensities were measured within the region of interest (ROI). Optical images were performed using a Caliper Life Sciences imaging system (excitation = 630 nm, emission = 700 nm).

### Apoptosis assay

Labeled NK-92 cell toxicity was measured by CCK-8 assay in 4T1 and MCF-7 target cells. 4T1 cells were seeded into 6-well plates at 5 × 10^5^ cells per well in DMEM containing 10% FBS and incubated for 24 h (37 °C, 5% CO_2_). Then, NK-92 cells labeled with Fe_3_O_4_-PEG-CD56@Ce6 or NK-92 cells labeled with Fe_3_O_4_-PEG-CD56/Avastin@Ce6 were coincubated with the target cells for 24 h at 37 °C. The cells were collected in Eppendorf tubes, and after washing twice with PBS, the cells were stained with an Annexin V-FITC Apoptosis Detection Kit. The staining procedure was conducted at room temperature in the dark. The stained cells were analyzed by FCM.

### Western blot

4T1 and MCF-7 cells in 6-well plates at a density of 5 × 10^4^ cells per well were treated as indicated. Proteins were isolated from the cells by utilizing RIPA buffer with the help of a Protease/Phosphatase Inhibitor Cocktail. A modified BCA Protein Assay Kit was used to assess protein purity. The protein samples were then subjected to SDS-PAGE and transferred onto PVDF membranes (Millipore, Bedford, MA). The primary antibodies listed below were utilized to probe the specific target proteins: anti-Bax, anti-Bcl-2, anti-cleaved caspase-3, anti-cleaved PARP and anti-GAPDH, all of which were purchased from Cell Signaling Technology. After incubation with the secondary antibodies (CST), the target bands were developed with an EasyBlot ECL kit.

### Establishment of the mouse breast cancer model

Animal experiments and animal care were conducted according to protocols approved by the institutional committee. Female 5-week-old BALB/c nude mice were purchased from Shanghai SLAC Laboratory Animal Co. (Shanghai, China) and maintained in a specific pathogen-free (SPF) environment. The tumor-bearing mouse model was established by subcutaneously injecting 100 µL of 4T1 cell suspension containing 1 × 10^6^ cells into each mouse in the right mammary fat pad.

### In vivo MR and optical dual-modal imaging

MR and optical dual-modal imaging and biodistribution studies were performed when the tumor diameters were 5–10 mm. NK-92 cells labeled with Fe_3_O_4_-PEG-CD56/Avastin@Ce6 were injected into tumor-bearing mice via the tail vein. In vivo MRI was carried out using a 3.0 T MR system with a high-resolution animal coil before injection and 2 h, 4 h, 6 h, 12 h and 24 h after intravenous injection. The T2WI used the following scanning parameters: TR = 2000 ms, TE = 97 ms, flip angle = 90°, FOV = 60 mm×80 mm, slice thickness = 2 mm, and slice gap = 0 mm. Optical images were obtained using a Caliper Life Sciences imaging system before injection and 6 h, 12 h and 24 h after intravenous injection (excitation = 630 nm, emission = 700 nm).

### In vivo antitumor effects

The tumor-bearing mice were randomly divided into four groups and treated with PBS, NK-92 cells (2 × 10^7^ cells/0.1 mL of PBS), NK + NPs and NK + NPs + L. The NK + NPs + L group was irradiated with a laser for 15 min at 6 h, 12 h and 24 h after injection. The tumor volumes and mouse body weights were recorded daily. The longest (a) and shortest (b) diameters of the tumors were measured with a digital Vernier caliper to determine the tumor volumes. After 15 days, all animals were sacrificed and the main organs were excised for H&E staining.

### Statistical analysis

All quantitative data are expressed as the mean ± standard deviation (SD). SPSS 25.0 statistical software (SPSS Institute, Cary, North Carolina, USA) was used for statistical analyses. Data among multiple groups were compared using one-way ANOVA followed by the least significant difference (LSD) test. *P* values < 0.05 were considered statistically significant.

## Results and discussion

### Characterization of Fe_3_O_4_-PEG-CD56/Avastin@Ce6

**Fig. 2:**
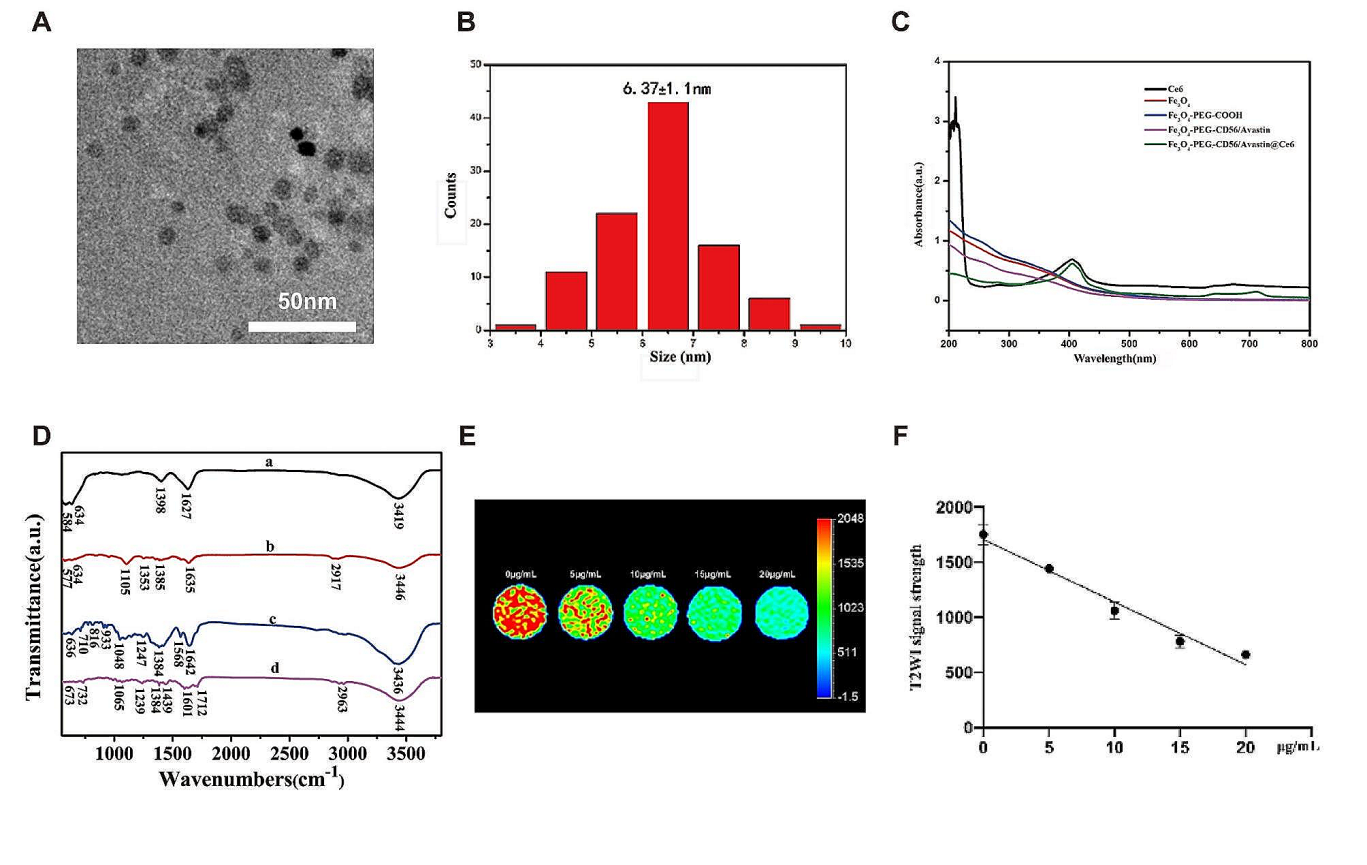
Characterization of the Fe_3_O_4_-PEG-CD56/Avastin@Ce6 NPs. (**A**) TEM image of the synthesized Fe_3_O_4_-PEG-CD56/Avastin@Ce6 NPs. (**B**) Size distribution of the Fe_3_O_4_-PEG-CD56/Avastin@Ce6 NPs determined from the TEM images. (**C**) UV-visible absorption spectrum. (**D**) FTIR spectrum of samples (a: Fe_3_O_4_ NPs, b: Fe_3_O_4_-PEG NPs, c: Fe_3_O_4_-PEG-CD56/Avastin NPs, and d: Fe_3_O_4_-PEG-CD56/Avastin@Ce6 NPs). (**E**) T2-weighted MR images of the Fe_3_O_4_-PEG-CD56/Avastin@Ce6 nanoprobes in aqueous solutions at various Fe concentrations. (**F**) T2 relaxation rates of the Fe_3_O_4_-PEG-CD56/Avastin@Ce6 nanoprobes

TEM and histogram images showed the size distribution of the formed Fe_3_O_4_-PEG-CD56/Avastin@Ce6 nanoprobes. The shape of Fe_3_O_4_-PEG-CD56/Avastin@Ce6 nanoprobes was spherical as observed from TEM image (Fig. 2A). The average particle size of the Fe_3_O_4_-PEG-CD56/Avastin@Ce6 nanoprobes was measured to be 6.37 nm (Fig. 2B). The hydrodynamic size of the nanoprobes was consistent to the particle size measured by TEM and the polydispersity index (PDI) of nanoprobes was less than 0.2 (Fig. S1). Moreover, both the hydrodynamic sizes and PDIs did not obviously change after 7 days of storage, which indicated the good colloid stability of nanoprobes. After two weeks, no obvious changes in the morphology or diameter of the NPs were observed, indicating that the nanomaterial was stable in structure (Fig. S2). Various characterizations were undertaken to confirm the successful synthesis of the Fe_3_O_4_-PEG-CD56/Avastin@Ce6 nanoprobes. UV-vis absorption spectroscopy of the Fe_3_O_4_-PEG-CD56/Avastin@Ce6 nanoprobes showed the distinct characteristic absorption peak of Ce6 at 405 nm, and those from PEG, the anti-CD56 antibody and Avastin between 200 and 300 nm (Fig. 2C). As shown by Fourier transform infrared (FTIR) spectroscopy (Fig. 2D), the peaks from the Fe_3_O_4_-PEG-CD56/Avastin@Ce6 nanoprobes (curve d in Fig. 2D) at 1105 nm and 2963 nm originated from the bending vibrations of the C-O-C bond and C-H bond of PEG. Moreover, the strong absorption peaks at 1601 nm and 1712 nm corresponded to the carboxyl stretching vibration of the amide group (-CO) and the bending vibration of the N-H bond, indicating successful conjugation of PEG, the anti-CD56 antibody and Avastin. T2-weighted MR images (T2WI) of the Fe_3_O_4_-PEG-CD56/Avastin@Ce6 nanoprobes at different Fe concentrations were obtained to evaluate the ability of the Fe_3_O_4_-PEG-CD56/Avastin@Ce6 nanoprobes to act as contrast agents. As shown in Fig. 2E, the signal intensity from the T2WI gradually decreased with increasing Fe concentration. After generating a plot with the Fe concentration as the abscissa and the T2 relaxation time as the ordinate, a linear relationship was found (Fig. 2F). The thermal stability and decomposition behavior of Fe_3_O_4_ were evaluated by thermogravimetric analysis (TGA) under a nitrogen atmosphere, as shown in Fig. S3. The weight loss from 100 to 400 °C corresponded to the removal of unstable oxygen-containing groups from the organic species. Further weight loss from 450 to 750 °C was related to the removal of the relatively stable oxygen-containing groups. When the temperature was above 750 °C, the oxygen-containing groups on the surface of Fe_3_O_4_ were completely removed. The total weight loss of 11.8% revealed that the surface of the synthesized Fe_3_O_4_ NPs contains a large number of carboxyl groups.

### Cell viability evaluation

**Fig. 3:**
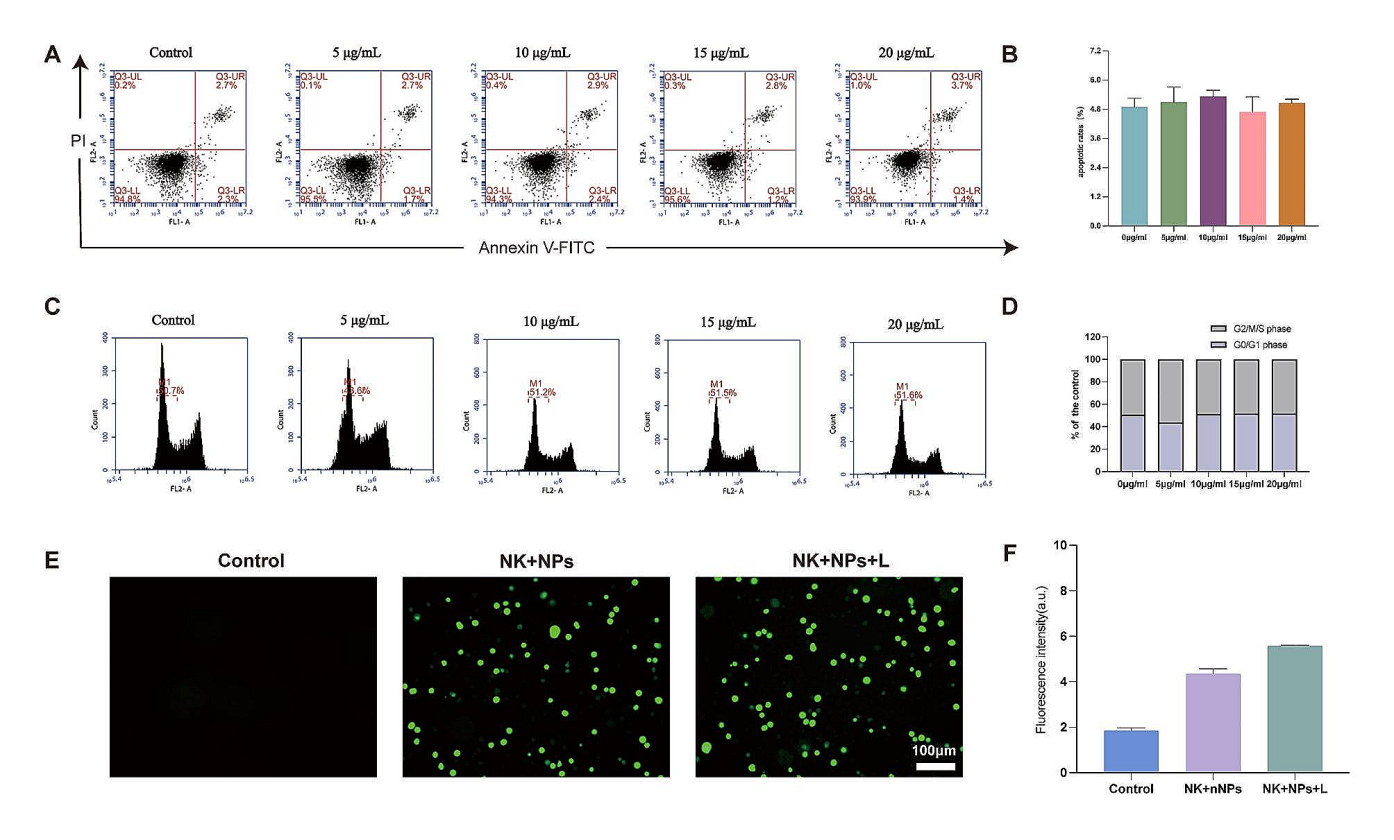
FCM analysis of Fe_3_O_4_-PEG-CD56/Avastin@Ce6 NP-labeled human NK-92 cells. (**A**) Rate of cell apoptosis via FCM analysis. (**B**) Statistical analysis of cell apoptosis in different groups. (**C**) Percentage of cells at different stages of the cell cycle by FCM analysis. (**D**) Statistical analysis of cell cycle in different groups. (**E**) Fluorescence images of NK cells with generation of ROS. (**F**) Statistical analysis of ROS signal fluorescence intensity in different groups

To investigate the effects of Fe_3_O_4_-PEG-CD56/Avastin@Ce6 NPs on the physiological characteristics of NK-92 cells, cell apoptosis and cell cycle analyses were performed. The cell apoptosis rate was measured by flow cytometry (FCM). The NK-92 cells in different treatment groups exhibited the same number of early and late apoptotic cells (Fig. 3A). These findings suggested that even high concentration of Ce6 (20 µg/mL) did not induce NK-92 cell apoptosis (Fig. 3B). As shown in Fig. 3C, D, the cell cycle did not significantly change at different Ce6 concentrations.

To further assess the effect of nanoprobes on NK cells during laser, the cell viability of NK-92 cells after treatment and laser irradiation was measured via cell counting kit-8 (CCK-8) assay. The cell viability gradually decreased with increasing Ce6 concentration during laser (Fig. S4). More than 60.0% of the cells survived at the highest vector concentration after 48 h. The above results indicated that laser irradiation had little effect on NK-92 cell viability. ROS generation was measured in NK + NPs and NK + NPs + L. Interestingly, both nanoprobes treated cells showed some extent of green fluorescence intensity (Fig. 3E). The NK + NPs + L treated cells exhibited much higher green fluorescence intensity compared with the other groups (Fig. 3F). Taken together, these results suggested that these NPs possessed good biocompatibility within this concentration range and could be safely used for further study.

### Cellular uptake evaluation

**Fig. 4:**
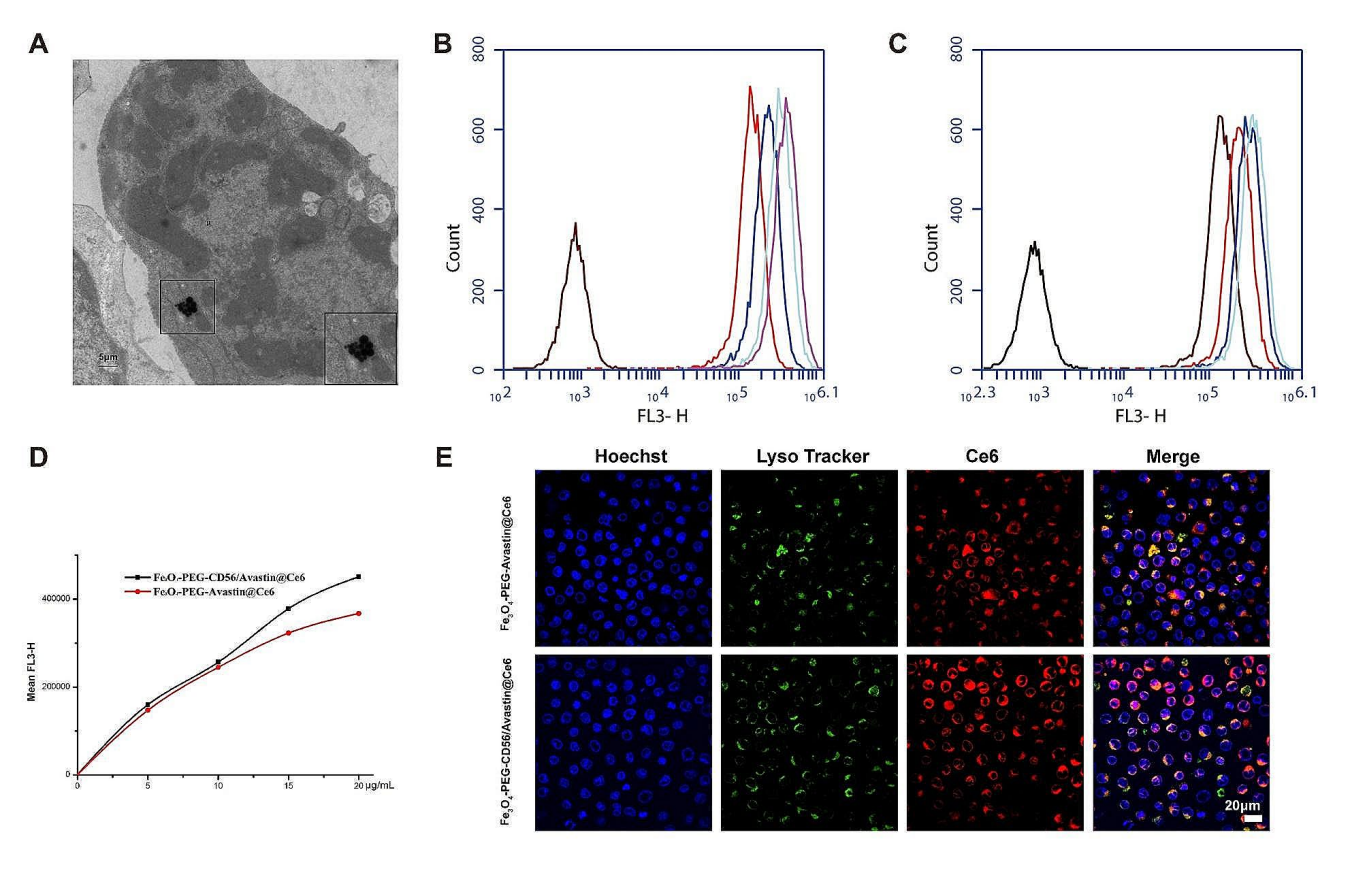
Cellular uptake of the nanoprobes. (**A**) TEM image of NK-92 cells after 24 h of incubation with the Fe_3_O_4_-PEG-CD56/Avastin@Ce6 nanoprobes. (**B**) FCM analysis of uptake Fe_3_O_4_-PEG-Avastin@Ce6 probes for NK-92 cells. (**C**) FCM analysis of uptake Fe_3_O_4_-PEG-CD56/Avastin@Ce6 probes for NK-92 cells. (**D**) Quantitative analysis of FCM for NK-92 cells after different treatments. (**E**) CLSM observation of cellular uptake of nanoprobes by NK-92 cells after treatments and lysosomal staining (scale bar = 20 μm)

TEM was used to investigate the internalization of the Fe_3_O_4_-PEG-CD56/Avastin@Ce6 NPs into NK-92 cells (Fig. 4A). The TEM images of the NK-92 cells revealed that the NPs appeared with high electron densities inside the cell cytoplasm. Inductively coupled plasma (ICP) analysis also indicated the uptake of NPs by NK-92 cells after incubation (Table S1). The cellular uptake of the nanoprobes with and without the anti-CD56 antibody was evaluated in vitro using FCM (Fig. 4B-D). As the Ce6 concentration increased, the average value of the fluorescence intensity of NK-92 cells increased. Although the numbers of NPs in each cells were not consistent, the mean fluorescence intensity value of the cells incubated with Fe_3_O_4_-PEG-CD56/Avastin@Ce6 was significantly higher than that of the cells treated with Fe_3_O_4_-PEG-Avastin@Ce6 at the same Ce6 concentration. This result indicated that the anti-CD56 antibody increased the uptake of these NPs, which would promote cancer therapy effectiveness. Afterwards, CLSM was employed to evaluate the cell uptake. Cellular colocalization staining revealed that the Fe_3_O_4_-PEG-CD56/Avastin@Ce6 successfully entered into lysosome (Fig. 4E). The fluorescence intensity for Fe_3_O_4_-PEG-CD56/Avastin@Ce6-treated cells was higher than that of Fe_3_O_4_-PEG-Avastin@Ce6-treated cells (Fig. S5A). The locations of red fluorescence signals of the Fe_3_O_4_-PEG-CD56/Avastin@Ce6 and Fe_3_O_4_-PEG-Avastin@Ce6 were close to that of blue lysosomal staining signals, indicating that the NPs could be easily internalized by NK-92 cells via lysosome (Fig. S5B-C).

### MR and optical imaging in vitro in NK-92 cells

**Fig. 5:**
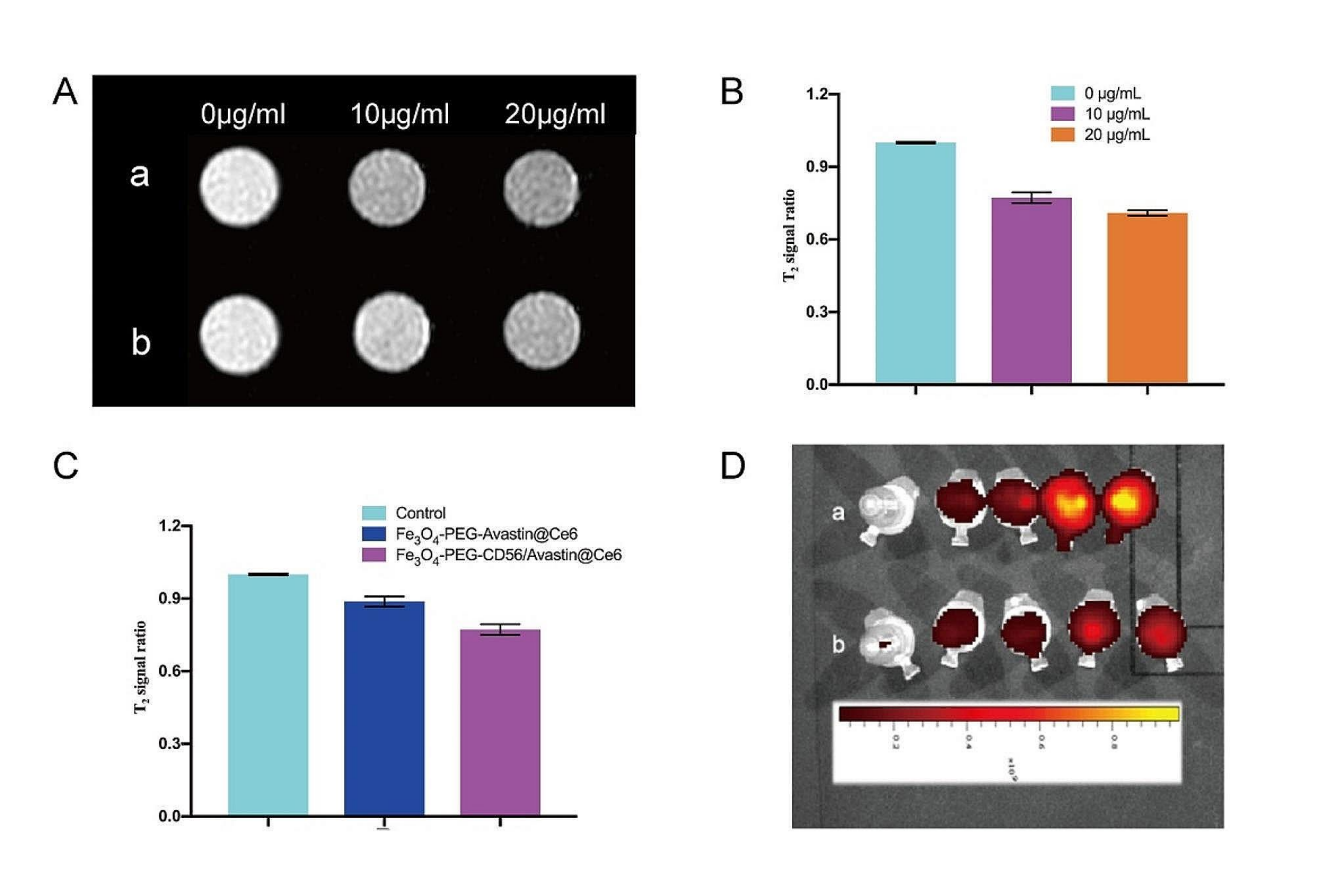
MR and optical imaging in vitro in NK-92 cells. (**A**) MRI of NK-92 cells labeled with nanoprobes (a: Fe_3_O_4_-PEG-CD56/Avastin@Ce6-labeled NK-92 cells; b: Fe_3_O_4_-PEG-CD56/Avastin@Ce6-labeled NK-92 cells). (**B**) Comparison of the T2WI signal ratio between the different concentrations of Ce6 determined by MRI. (**C**) Comparison of the T2WI signal ratio between the different nanoprobes determined by MRI. (**D**) Fluorescence images of NK-92 cells labeled with nanoprobes (a: Fe_3_O_4_-PEG-CD56/Avastin@Ce6; b: Fe_3_O_4_-PEG- Avastin@Ce6)

To determine the ability of Fe_3_O_4_-PEG-CD56/Avastin@Ce6 to target NK-92 cells, the T2 signal intensity for NK-92 cells after incubation with NPs at different concentrations of Ce6 was measured (Fig. 5A). The results showed that the T2 signal intensity decreased significantly as the Fe_3_O_4_ concentration in the NPs increased. The MRI signal intensity of the NK-92 cells labeled with Fe_3_O_4_-PEG-CD56/Avastin@Ce6 decreased more significantly than that of the cells labeled with Fe_3_O_4_-PEG-Avastin@Ce6 at the same Ce6 concentration (10 µg/mL) (Fig. 5B-C). Furthermore, optical imaging was performed to verify the targeting effect. As shown in Fig. 5D, NK-92 cells incubated with the Fe_3_O_4_-PEG-CD56/Avastin@Ce6 nanoprobes exhibited much stronger fluorescence intensity than cells treated with Fe_3_O_4_-PEG-Avastin@Ce6. These results suggested that Fe_3_O_4_-PEG-CD56/Avastin@Ce6 displayed a higher targeting ability than Fe_3_O_4_-PEG-Avastin@Ce6.

### In vitro therapeutic efficacy evaluation

**Fig. 6:**
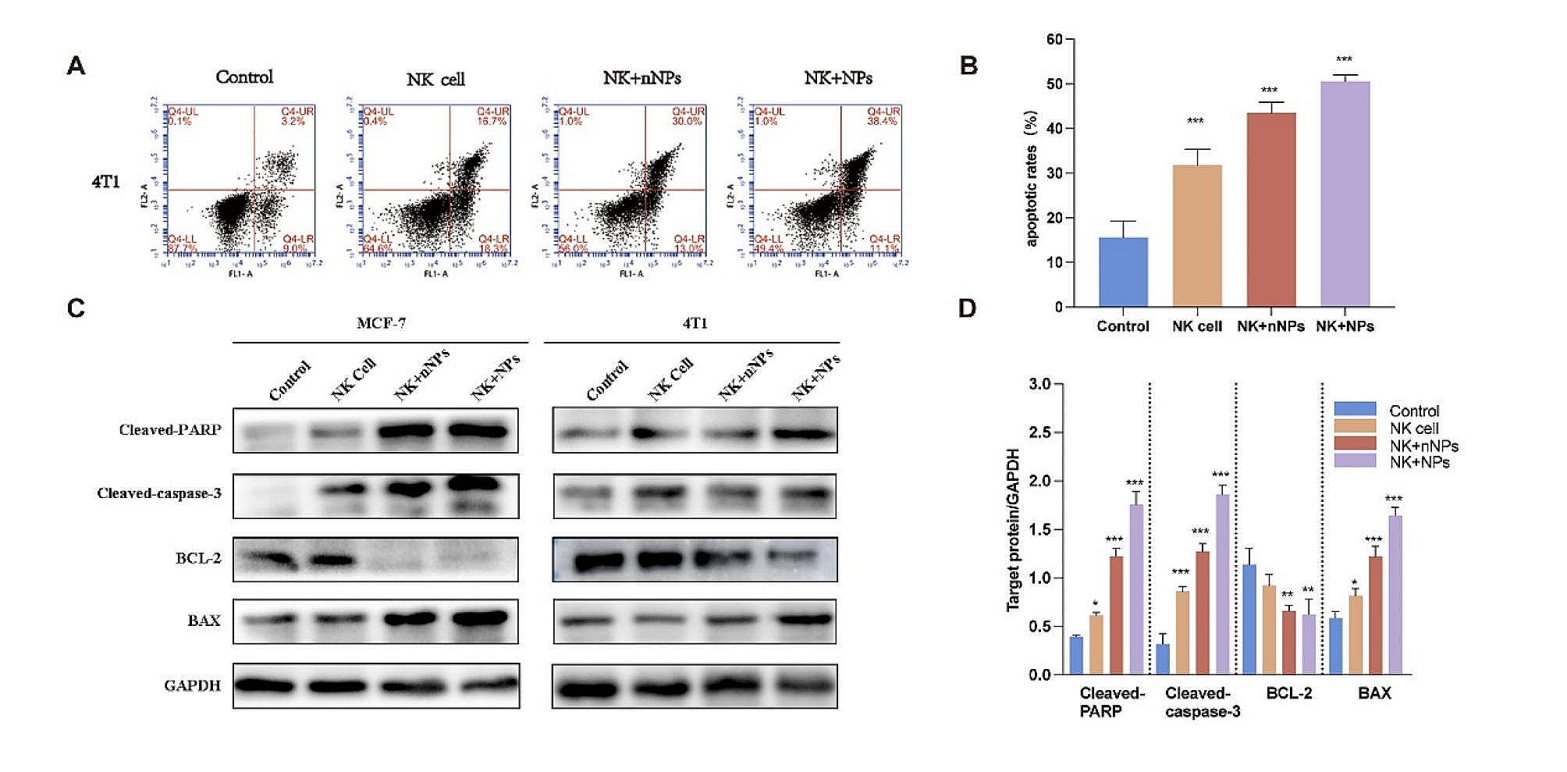
In vitro therapeutic efficacy. (**A**) Apoptosis analysis of 4T1 cells after different treatments by FCM analysis. (**B**) Statistical analysis of cell apoptosis with different group by FCM analysis. (**C**) Expression of related apoptotic proteins in breast cancer cells after different treatments by western blot analysis. (**D**) Statistical analysis of cell apoptosis by western blot analysis

The in vitro therapeutic efficacy was evaluated by FCM using MCF-7 and 4T1 breast cancer cells. Figure 6A and Fig. S6 indicated that the NK + NPs were the most cytotoxic to the 4T1 and MCF-7 cells compared to the other groups. The apoptotic activity of the NK + NPs was higher than that of NK-92 cells alone or NK-92 cells loaded with Fe_3_O_4_-PEG-CD56@Ce6 (Fig. 6B).

Furthermore, we investigated the effects of NK + NPs on the expression of apoptotic markers in breast cancer cells. Western blotting was performed to identify the expression of Bax, Bcl-2, cleaved caspase-3 and cleaved PARP. In the NK + NP group, Bcl-2 was downregulated and Bax, cleaved caspase-3 and cleaved PARP were upregulated (Fig. 6C-D). Collectively, these results indicated that NK + NPs more successfully induced tumor cell apoptosis and Avastin improved the effectiveness of cancer therapy.

### In vivo dual-modal imaging and cancer therapy evaluation

**Fig. 7:**
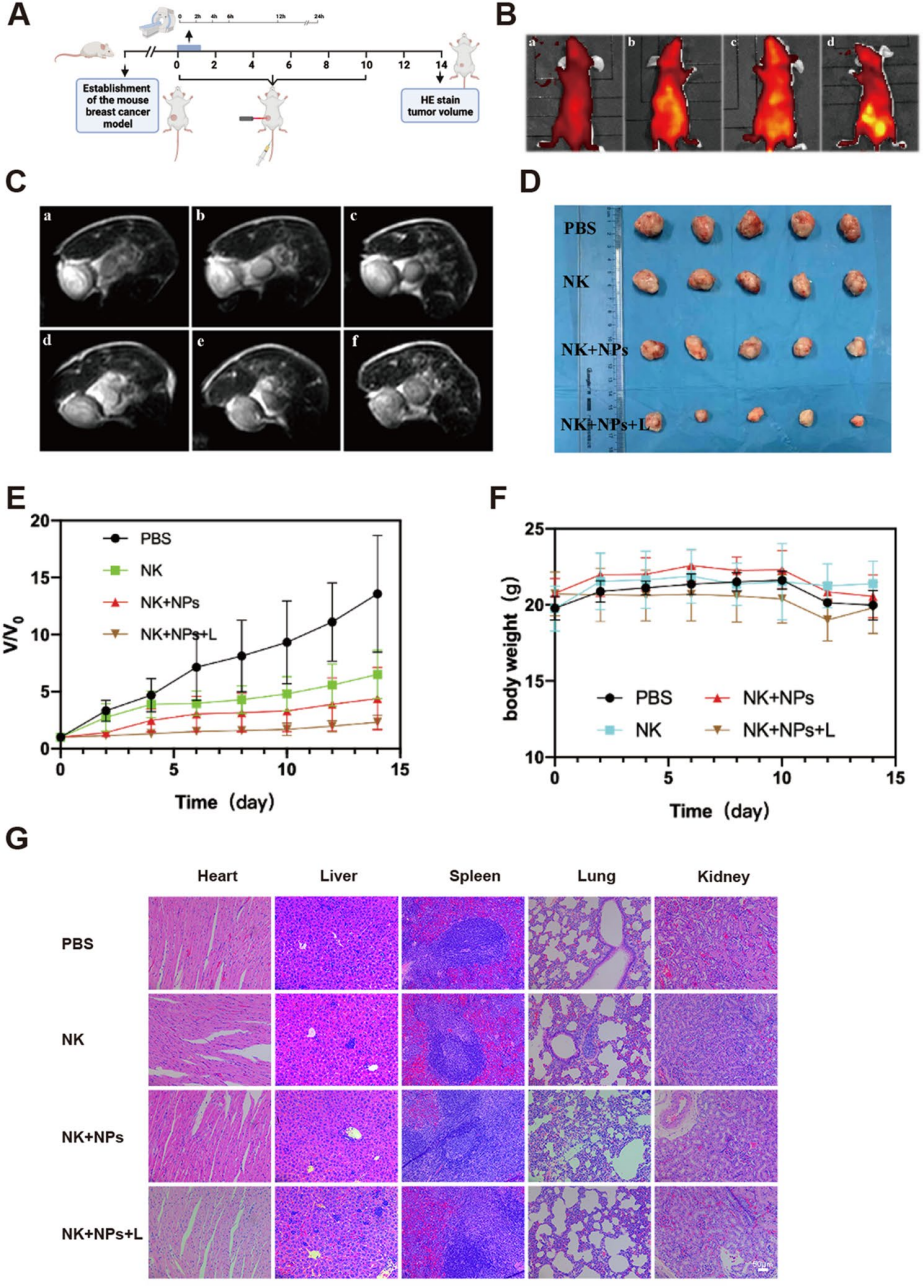
In vivo dual-modal imaging and cancer therapy. (**A**) schematic diagram of the in vivo experimental design. (**B**) In vivo fluorescence imaging of mice (a, b, c and d correspond to the fluorescence images before injection of NK-NPs into the tail vein and at 6 h, 12 h and 24 h after injection, respectively). (**C**) In vivo MRI of mice (a, b, c, d, e and f correspond to the MR images before injection of NK-NPs into the tail vein and at 2 h, 4 h, 6 h, 12 h, and 24 h after injection, respectively). (**D**) Photographs of the mouse tumors after various treatments. (**E**) Relative mouse tumor volume curves after 15 days of different treatments. (**F**) Body weight curves of the mice after 15 days of treatments. (**G**) Representative H&E staining images of the hearts, lungs, livers, spleens and kidneys of mice after 15 days of treatments

The pharmaceutic kinetic of NP-labeled NK-92 cells was evaluated after injection into mice. As shown in Fig. S7, the pharmaceutic kinetic of NP-labeled NK-92 cells was similar to that of NK-92 cells, suggesting that NK-92 cells could still effectively circulate in the blood after phagocytosis of NPs. As shown in Fig. 7A, a schematic diagram of the in vivo experimental design was given. To investigate the tumor-specific targeting properties of the NK + NPs, optical imaging was applied to monitor the intrinsic near infrared (NIR) fluorescence signal of Ce6 in the nanoprobes. When the tumor nodules of nude mice reached a volume of 60–80 mm [3], NK + NPs were intravenously injected for the real-time detection of drug distribution in vivo. Figure 7B showed the changes in the fluorescence signals in the nude mice before injection and 6 h, 12 h and 24 h post-injection. The fluorescence signal at the tumor site gradually increased with time, which implied that the NK + NPs could target the tumors. To further investigate whether NK + NPs could be used as an effective imaging agent, MRI was carried out before and 2 h, 4 h, 6 h, 12 h and 24 h after intravenous injection (Fig. 7C). The MR signal in the tumor tissues was found to gradually decrease over time. This result indicated that the NK + NPs localized in the tumor, which was consistent with the fluorescence imaging results. These results indicated that the NPs could target tumors, thus contributing to efficient innate immune cell therapy and PDT.

Therapeutic efficacy was evaluated via 4T1 tumor xenografts in BALB/c nude mice. The experimental mice were divided into four groups: PBS, NK-92 cells, NK + NPs and NK + NPs + L. The changes in tumor volume were monitored over 15 days (Fig. 7D-E). Over the 15 days, all treated groups showed tumor growth inhibition compared to the PBS group, suggesting that NK-92 therapy majorly contributed to tumor suppression. Tumor growth in the NK + NP group showed a more efficient lag than that found in the NK-92 group. This finding indicated that the application of Fe_3_O_4_-PEG-CD56/Avastin@Ce6 may enhance the therapeutic effects by improving the local number of NK-92 cells in the tumor region. Notably, tumor growth in the NK + NPs + L group was significantly inhibited compared with the other three groups, illustrating the effectiveness of immunotherapy combined with PDT. These results demonstrated that the targeting method effectively enhanced the tumor treatment efficacy.

To confirm and investigate the potential toxicity of NK + NPs in mice, the changes in the weights of the tumor-bearing mice were monitored as an indicator of poisonous side effects. As shown in Fig. 7F, no noticeable changes in mouse weight were observed in any of the four groups. On day 15, the major organs and tissues (heart, liver, spleen, lungs, and kidneys) were collected from the mice in the four groups and sliced, stained with hematoxylin and eosin (H&E) according to the standard protocol, and then observed by microscopy. Notably, no pathological abnormalities or other conditions were observed after histological examination of the major organ tissues from the mice (Fig. 7G). Thus, no noteworthy signs of toxic side effects from the NK + NPs + L were observed in vivo, as further uncovered by histological examination after PDT treatment.

## Conclusion

In summary, Fe_3_O_4_-PEG-CD56/Avastin@Ce6 NPs were synthesized and shown to be taken up by NK-92 cells. Within a certain concentration range, the physiological properties of the NK-92 cells remained unaffected by the NPs, and their ability to kill the target cells was significantly enhanced when the NK-92 cells were labeled with Fe_3_O_4_-PEG-CD56/Avastin@Ce6. 4T1 xenograft tumors in mice were detected by MRI and optical imaging after a single intravenous administration of the nanoprobes. In addition, the antitumor therapeutic efficiency of NK-92 cells labeled with Fe_3_O_4_-PEG-CD56/Avastin@Ce6 was significantly enhanced by PDT under laser irradiation. These findings strongly suggest that the designed multifunctional Fe_3_O_4_-PEG-CD56/Avastin@Ce6 nanocomposites can be potential nanoprobes for targeting innate immune cells, administering PDT and MR/optical dual-mode imaging. This study also showed that it is possible to observe and assess the curative effects of immunotherapy through imaging technologies in patients with breast cancer without the need for invasive, damaging or time-consuming methods to optimize and individualize breast cancer therapy for future clinical applications.

## Electronic supplementary material

Below is the link to the electronic supplementary material.

Supplementary Material 1

## Data Availability

All relevant data are within the manuscript and supplement files.
