# Correction: NK-92 cells labeled with Fe_3_O_4_-PEG-CD56/Avastin@Ce6 nanoprobes for the targeted treatment and noninvasive therapeutic evaluation of breast cancer

**DOI:** 10.1186/s12951-026-04332-2

**Published:** 2026-04-10

**Authors:** Jingge Lian, Meng Li, Meng Duan, Xiaoqian Sun, Zilin Wang, Xinyu Guo, Jingchao Li, Guo Gao, Kangan Li

**Affiliations:** 1 Department of Radiology, Songjiang Hospital Affiliated to Shanghai Jiaotong University School of Medicine, Shanghai, 201600 P.R. China; 2 Department of Radiology, Peking University Third Hospital, Beijing, 100191 China; 3 State Key Laboratory for Modification of Chemical Fibers and Polymer Materials, College of Biological Science and Medical Engineering, Donghua University, Shanghai, 201620 China; 4 Department of Instrument Science and Technology, School of Electronic Information and Electrical Engineering, Shanghai Jiao Tong University, Shanghai, 200240 China; 5 Department of Immunology, School of Cell and Gene Therapy, Songjiang Research Institute, Shanghai Jiao Tong University School of Medicine, Shanghai, 201600 P.R. China

**Correction: Journal of Nanobiotechnology (2024)** **22: 313**

10.1186/s12951-024-02599-x

The author regret an error in the presentation of images in the original publication. In Fig. 7G, the H&E staining image of liver in the PBS group was mistakenly used and was an inadvertent duplication of liver image in NK + NPs+L group. This error occurred due to a mistake during the data organization and figure assembly. The authors declare that this correction does not affect the validity of the original discussion, interpretation, and conclusion presented in this paper. For completeness and transparency, the old incorrect and correct versions are displayed below.

Incorrect Fig. 7.

**Fig. 7:**
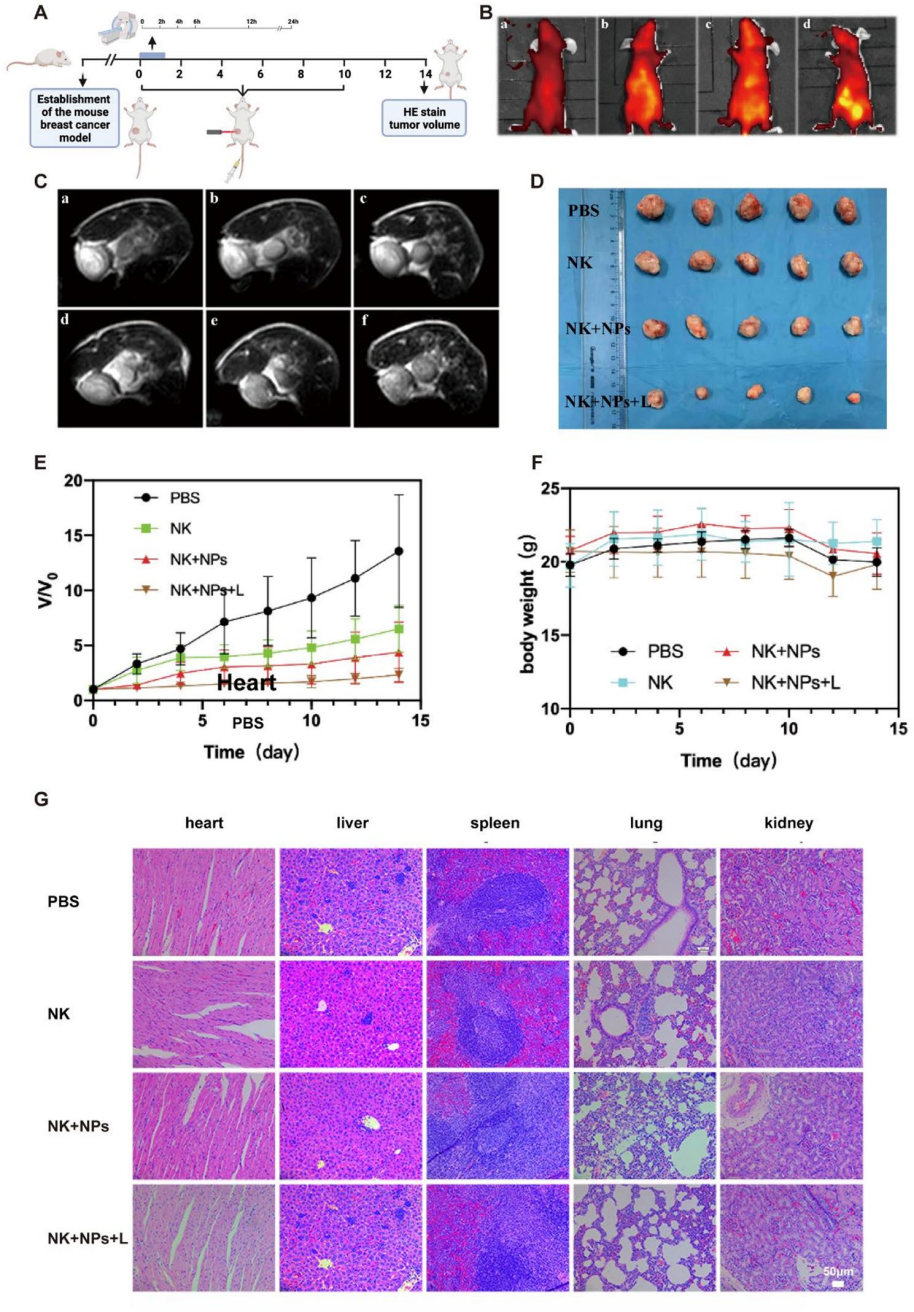
In vivo dual-modal imaging and cancer therapy. **(A)** schematic diagram of the in vivo experimental design. **(B)** In vivo fluorescence imaging of mice: a, b, c and d correspond to the fluorescence images before injection of NK-NPs into the tail vein and at 6 h, 12 h and 24 h after injection, respectively. **(C)** In vivo MRI of mice: a, b, c, d, e and f correspond to the MR images before injection of NK-NPs into the tail vein and at 2 h, 4 h, 6 h, 12 h, and 24 h after injection, respectively. **(D)** Changes in the sizes of the nude mouse tumors after various treatments. **(E)** Relative mouse tumor volume curves after 15 days of different treatments. **(F)** Body weight curves of the mice after 15 days of treatment. **(G)** Representative H&E staining images of the hearts, lungs, livers, spleens and kidneys of mice after 15 days of treatment

Correct Fig. 7.

**Fig. 7:**
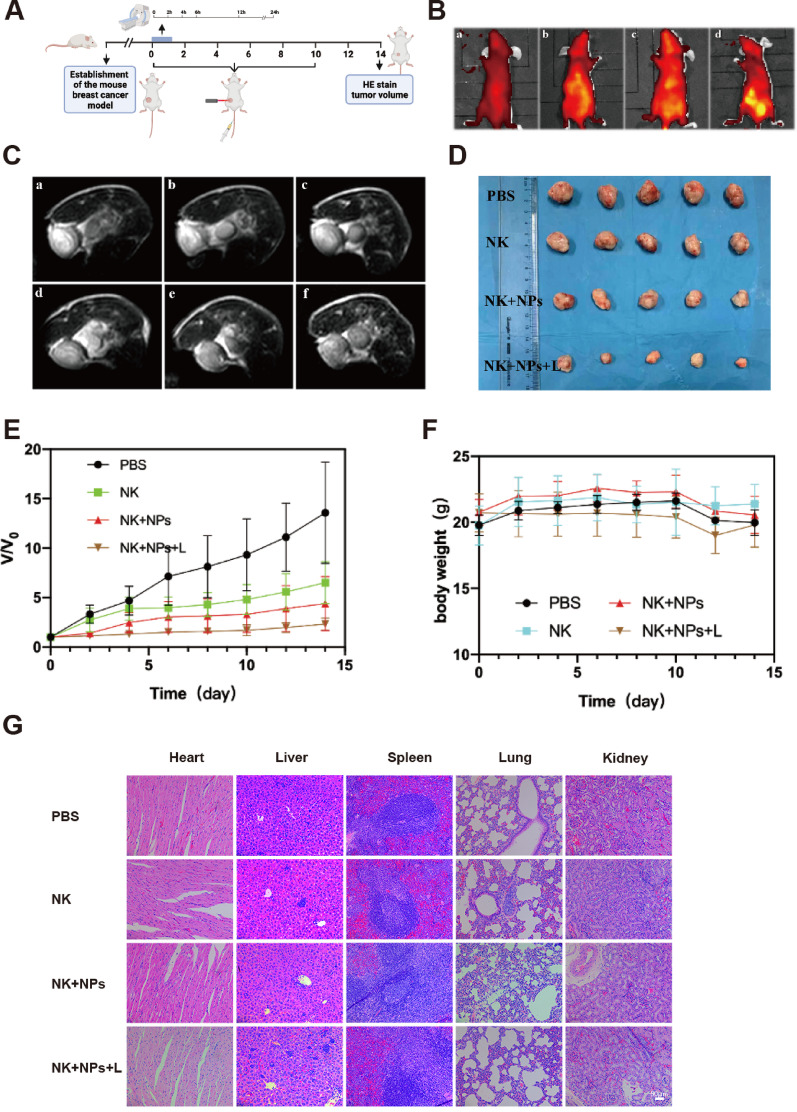
In vivo dual-modal imaging and cancer therapy. **(A)** schematic diagram of the in vivo experimental design. **(B)** In vivo fluorescence imaging of mice: a, b, c and d correspond to the fluorescence images before injection of NK-NPs into the tail vein and at 6 h, 12 h and 24 h after injection, respectively. **(C)** In vivo MRI of mice: a, b, c, d, e and f correspond to the MR images before injection of NK-NPs into the tail vein and at 2 h, 4 h, 6 h, 12 h, and 24 h after injection, respectively. **(D)** Changes in the sizes of the nude mouse tumors after various treatments. **(E)** Relative mouse tumor volume curves after 15 days of different treatments. **(F)** Body weight curves of the mice after 15 days of treatment. **(G)** Representative H&E staining images of the hearts, lungs, livers, spleens and kidneys of mice after 15 days of treatment

The original article has been corrected.

