# Supplementary material for: NK-92 cells labeled with Fe3O4-PEG-CD56/Avastin@Ce6 nanoprobes for the targeted treatment and noninvasive therapeutic evaluation of breast cancer
Source: J Nanobiotechnology. 2024 Jun 5;22:313. doi: 10.1186/s12951-024-02599-x (PMC11151526; doi:10.1186/s12951-024-02599-x)
Supplement: Supplementary file 1 — Supplementary Material 1 [file 12951_2024_2599_MOESM1_ESM.docx]

**NK-92 cells labeled with Fe_3_O_4_-PEG-CD56/Avastin@Ce6 nanoprobes for the targeted treatment and noninvasive therapeutic evaluation of breast cancer**

Jingge Lian^1,2,#^, Meng Li^3,#^, Meng Duan^4,#^, Yaqian Sun^1,5^, Zilin Wang^1^, Xinyu Guo^1^, Jingchao Li^3*^, Guo Gao^4*^, Kangan Li^1*^

^1^ Department of Radiology, Songjiang Hospital Affiliated to Shanghai Jiaotong University School of Medicine, Shanghai 201600, P.R. China

^2^ Department of Radiology, Peking University Third Hospital, Beijing 100191, China

^3^ State Key Laboratory for Modification of Chemical Fibers and Polymer Materials, College of Biological Science and Medical Engineering, Donghua University, Shanghai 201620, China

^4^ Department of Instrument Science and Technology, School of Electronic Information and Electrical Engineering, Shanghai Jiao Tong University, Shanghai 200240, China

^5^ Department of Immunology, School of Cell and Gene Therapy, Songjiang Research Institute, Shanghai Jiao Tong University School of Medicine, Shanghai 201600, P.R. China

^#^ Jingge Lian, Meng Li, and Meng Duan contributed equally to this work.

^*^Corresponding authors:

kangan.li@shsmu.edu.cn (Kangan Li), guogao@sjtu.edu.cn (Guo Gao), jcli@dhu.edu.cn (Jingchao Li)


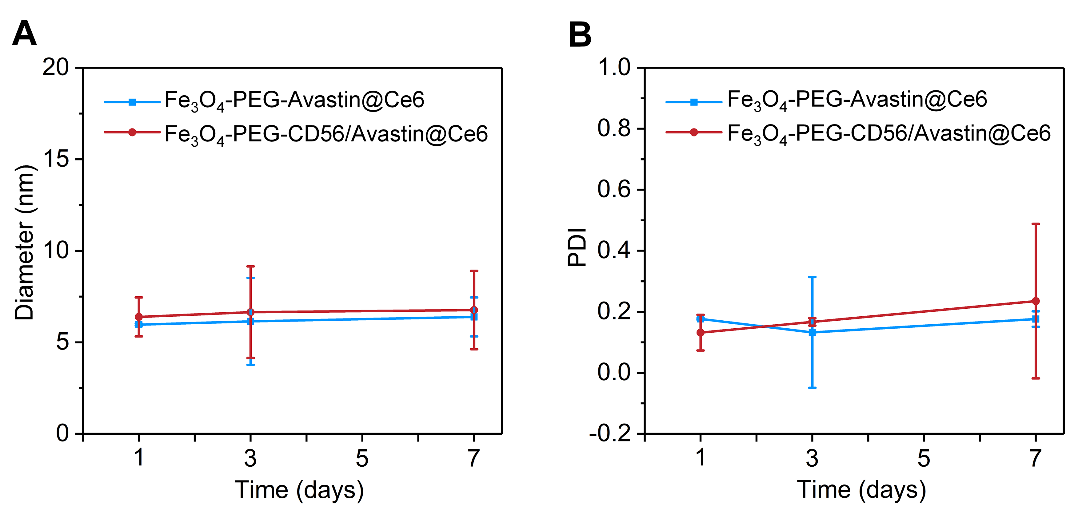


**Fig. S1.** (A) Hydrodynamic size and (B) PDI of nanoprobes after different days of storage.

**
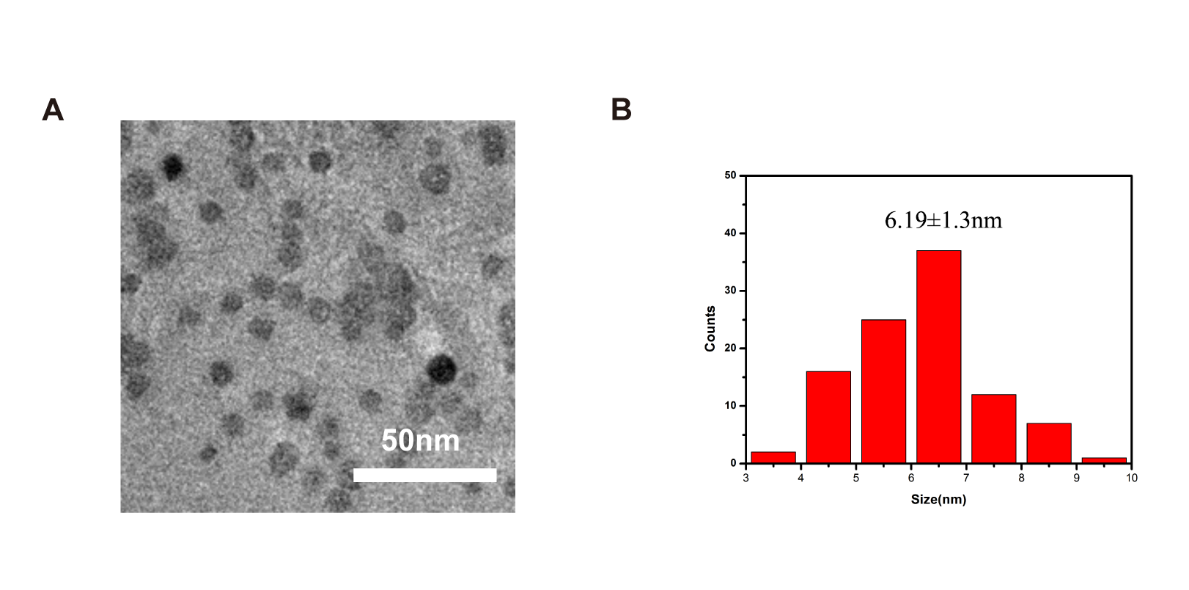
**

**Fig. S2.** (A) TEM image and (B) size distribution of Fe_3_O_4_-PEG-CD56/Avastin@Ce6 NPs after two weeks of storage in aqueous solution.

**

**

**Fig. S3.** TGA analysis of the Fe_3_O_4_ NPs.

**
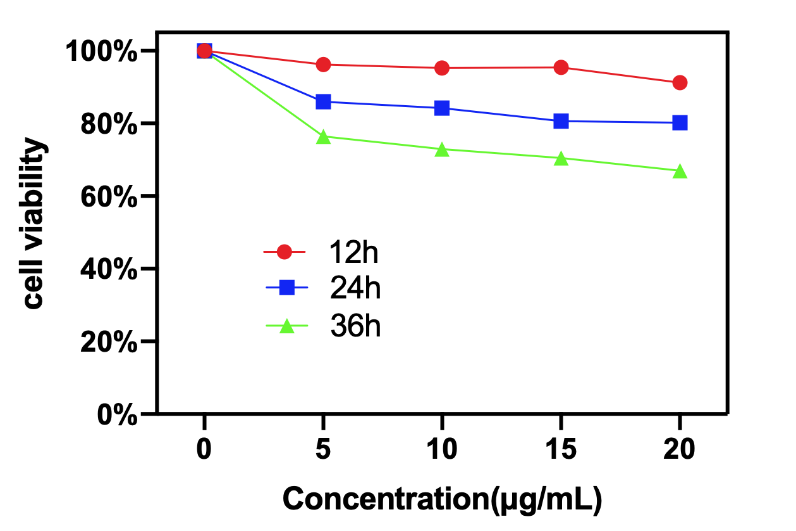
**

**Fig. S4.** Viability of NK-92 cells after incubation with various concentrations of Fe_3_O_4_-PEG-CD56/Avastin@Ce6 NPs.


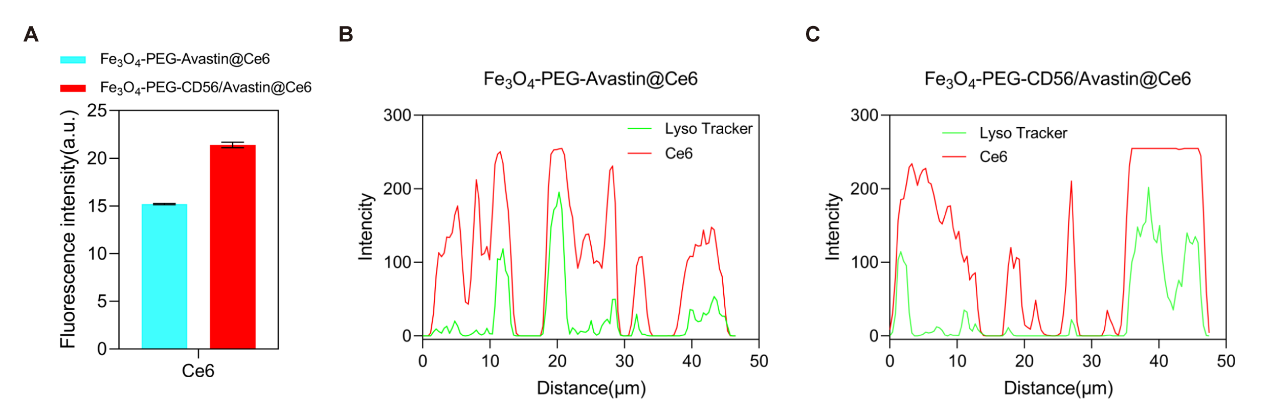


**Fig. S5.** (A) Fluorescence intensity of Fe_3_O_4_-PEG-CD56/Avastin@Ce6 and Fe_3_O_4_-PEG-Avastin@Ce6-treated cells. (B-C) Locations of red fluorescence signal analysis.

**
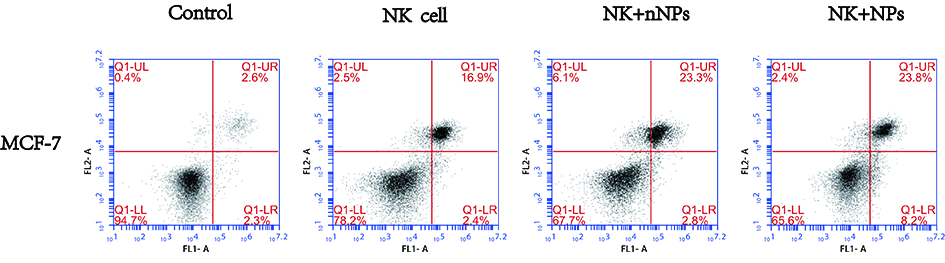
**

**Fig. S6.** Viability of NK-92 cells after incubation with various concentrations of Fe_3_O_4_-PEG-CD56/Avastin@Ce6 NPs.


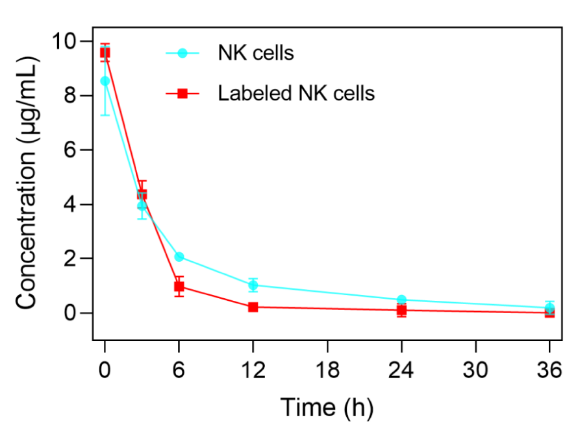


**Fig. S7.** The pharmaceutic kinetic analysis of NP-labeled NK-92 cells.

**Table S1.** ICP analysis of Fe level in NK-92 cells.

| Sample | Fe level in NK-92 cells |
| --- | --- |
| nNPs (0 μg/mL) | 0 μg/mL |
| nNPs (5 μg/mL) | 1.94 μg/mL |
| NPs (0 μg/mL) | 0 μg/mL |
| NPs (5 μg/mL) | 1.87 μg/mL |
